# Falciform Ligament Infarction: A Case Report and Review of the Literature

**DOI:** 10.7759/cureus.48361

**Published:** 2023-11-06

**Authors:** Divakar Hamal, André Fernandes, James Sagma, Mariana Rufino, Pravakar Hamal, Gabriele DiBenedetto

**Affiliations:** 1 Critical Care, King's College Hospital NHS Trust, London, GBR; 2 Trauma and Orthopaedics, Lewisham and Greenwich NHS Trust, London, GBR; 3 General Surgery, York & Scarborough Teaching Hospitals NHS Foundation Trust, Scarborough, GBR; 4 Medicine, Hospital de Faro, Quarteira, PRT; 5 General Surgery, University Hospital Llandough, Cardiff, GBR

**Keywords:** f-flat, case report, falciform ligament, fatty falciform ligament appendage torsion, fflat, falciform ligament infarction

## Abstract

The falciform ligament is a double peritoneal fold that separates the left and right hepatic lobes anatomically. Fatty-falciform ligament appendage torsion (F-FLAT) is defined as torsion of the extraperitoneal fat within the falciform ligament causing fat infarction, which is an uncommon surgical presentation, scarcely documented within the current literature. The objective of presenting this case report and reviewing the literature on F-FLAT is to discuss the clinical presentation, possible associated factors and management strategies in regard to this rare pathology.

A 72-year-old female patient presented to the emergency department with a seven-day history of epigastric pain, reduced appetite and nausea. On admission, the patient was stable and apyrexial with abdominal examination highlighting she was tender in her right upper quadrant and epigastric region. Due to the patient's unremitting abdominal pain despite appropriate analgesia, CT of the abdomen and pelvis (CTAP) with intravenous contrast was done and a diagnosis of F-FLAT was made. The patient was treated with antibiotics and analgesia, had a negative abdominal ultrasound (US) result and due to her symptoms settling by the second day of admission, she was discharged the same afternoon.

A literature review into falciform ligament infarction was conducted by two independent reviewers across four different databases: PubMed, Medline, Embase and the Cochrane Library. Search terms included “falciform ligament” OR “falciform” AND “infarction” (likewise with Medical Subject Headings, or MeSH, terms in the Cochrane Library). Eligibility criteria and our subsequent inclusion criteria were based on studies specifically discussing falciform ligament infarction and published in English. Study types were by majority case reports, but also included one literature review and a book source as well as two pictorial radiological reviews. All 13 patients presented with abdominal pain, but only 53% presented with raised infective/inflammatory markers. The majority of patients had abdominal US as a first-line investigation with 9 of 13 patients also having a CTAP with contrast, which classically showed fat stranding in the falciform ligament. Two patients had no evidence of any radiological investigation. Initially all cases were managed conservatively with non-steroidal anti-inflammatory drugs and analgesia, but in 62% of the cases (8/13), surgical intervention was needed due to unresolving abdominal pain. All eight of the excised falciform ligaments showed evidence of infarction and necrosis histologically.

In conclusion, F-FLAT is a relatively rare condition making it difficult to build higher level evidence studies. The current literature has revealed some evidence of incomplete and inconsistent data, for example, in the biochemical results and management techniques presented, yet contrast-enhanced CT seems moderately sensitive for detection in the reviewed literature. Though F-FLAT is rare and unfamiliar, it is vital we exclude common acute surgical pathologies that F-FLAT mimics and monitor for unsettling symptoms that could change the management trajectory.

## Introduction

The falciform ligament is a double peritoneal fold that separates the left and right hepatic lobes anatomically. Fatty-falciform ligament appendage torsion (F-FLAT) is defined as torsion of the extraperitoneal fat within the falciform ligament causing fat infarction, which is an uncommon surgical presentation, scarcely documented within the current literature [1]. It is a subset of the rare conditions known as the intraperitoneal focal fatty infarctions (IFFI) that also include omental infarctions, epiploic appendagitis, and other perigastric appendagitis [2,3]. Through this case report and literature review, we aim to portray this rare presentation’s clinical course, and discuss and scrutinise the existing data on this pathology [4].

## Case presentation

A 72-year-old female patient presented to the emergency department with a seven-day history of epigastric pain, reduced appetite and nausea. Pain was colicky in nature, severe in intensity and worse on food intake. There was no history of trauma or recent travel to foreign countries; there were no excess alcohol episodes or new changes in diet. She denied any other concerns or any systemic symptoms or significant co-morbidities.

**Figure 1:**
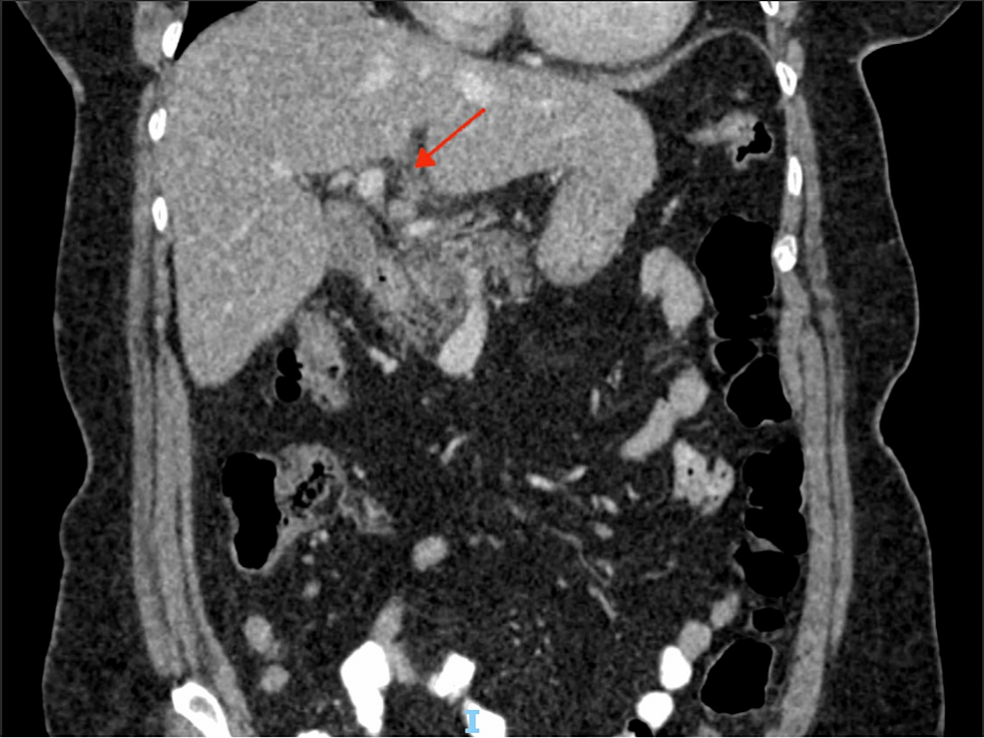
Coronal view of the CT scan of the abdomen and pelvis, showing fat stranding in the left subhepatic space centred around the falciform ligament.

**Figure 2:**
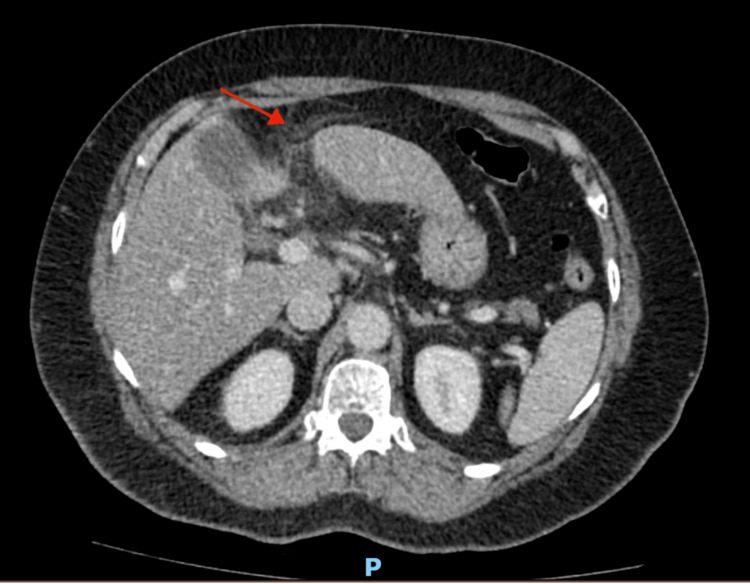
Axial view of the CT scan of the abdomen and pelvis, highlighting an inflammatory process centred on the falciform ligament.

On admission, the patient was stable and apyrexial. On abdominal examination, she was tender in her right upper quadrant and epigastric region. Clinically, Murphy’s sign was positive, but otherwise there were no overt signs of peritonitis. The laboratory results only showed a mild raise in the C-reactive protein (CRP; 60 mg/L) level, but otherwise all other tests (full blood count, amylase, liver function tests and urea and electrolytes) were within the normal range. An erect chest x-ray ruled out a pneumoperitoneum and also excluded any chest pathology. Given that the patient kept experiencing unremitting abdominal pain despite appropriate analgesia, a CT scan of the abdomen and pelvis (CTAP) with intravenous contrast was done, and with this, a diagnosis of F-FLAT was made (Figures 1, 2).

During the admission, the patient had intravenous antibiotics for two days (a 100 mg loading dose of tigecycline followed by a single dose of 50 mg tigecycline the next day), and analgesia in the form of opioids and antiemetics.

**Figure 3:**
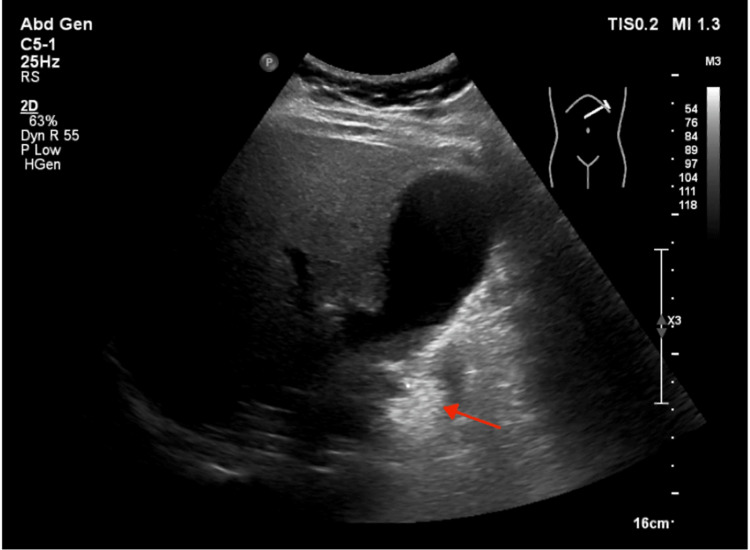
Axial grayscale ultrasound image with the arrow pointing to the small area of fat sparing

The next day, abdominal ultrasound (US) was done given her right upper quadrant (RUQ) pain and positive Murphy’s sign to rule out gallstone pathology or evidence of cholecystitis; this showed a small area of increased echotexture (fat sparing) surrounding the gallbladder fossa with no evidence of gallstones, nor any biliary tree/intrahepatic ductal dilatation (Figure 3). The patient’s symptoms had settled by the second day of admission and she was discharged the same afternoon.

## Discussion

Review methodology

**Figure 4:**
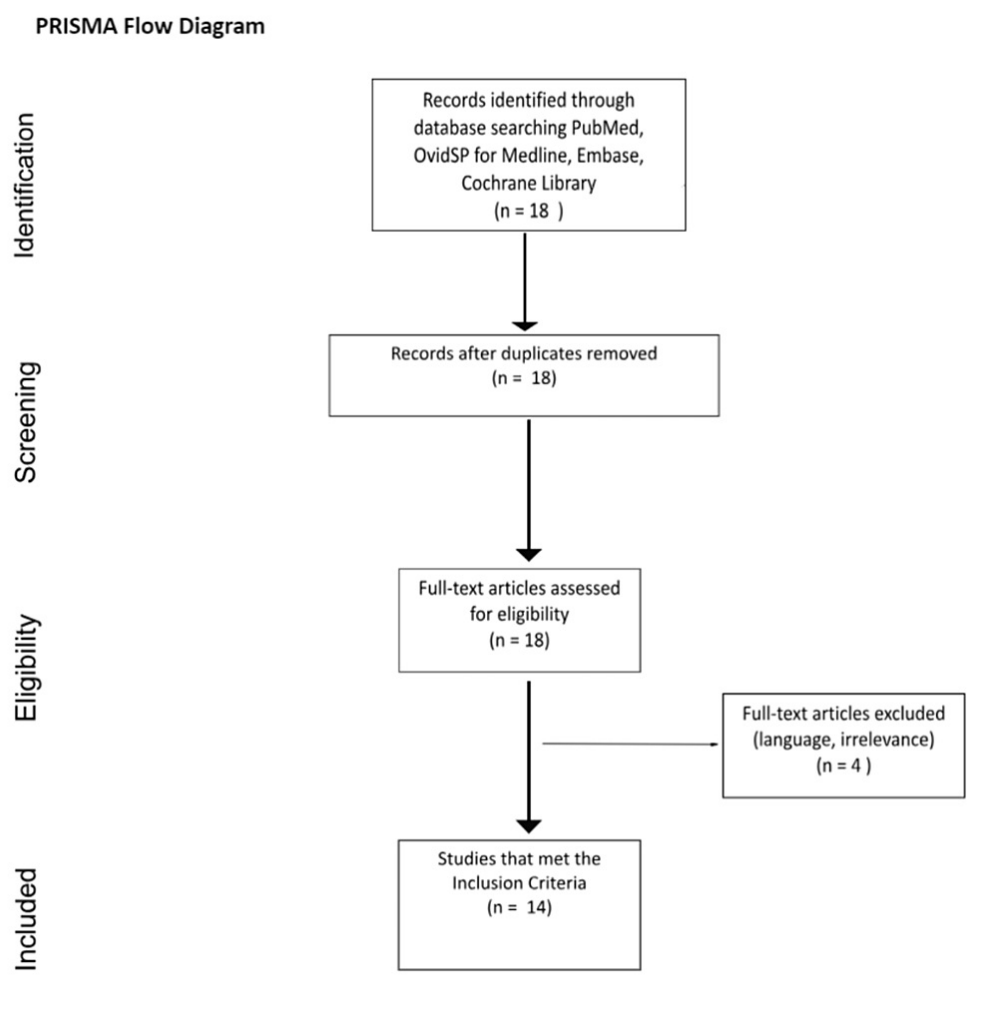
Preferred Reporting Items for Systematic Reviews and Meta-Analyses (PRISMA) flow diagram showing the study selection process

A literature review was conducted by two independent reviewers across four different databases: PubMed, Medline, Embase and the Cochrane Library in July 2022. Search terms included “falciform ligament” OR “falciform” AND “infarction” (likewise with Medical Subject Headings, or MeSH, terms in the Cochrane Library). Inclusion criteria were studies with adults only, studies specifically discussing falciform ligament infarction/F-FLAT and published in English. We also excluded patients less than 18 years of age. A Preferred Reporting Items for Systematic Reviews and Meta-Analyses (PRISMA) flow diagram is included to pictorially represent the inclusion and exclusion process (see Figure 4).

Outcome Measures

Primary outcome measures were classified as follows: population group, presenting complaint features, past medical and surgical history, biochemical markers, imaging used, conservative versus surgical management, follow-up and histological findings.

Results

The literature review initially identified a total of 18 studies that were analysed based on their title, abstract and full text. None of the studies found had duplicates or were inaccessible and so were further analysed to see if they fulfilled our eligibility criteria. This consequently led to the exclusion of four studies due to irrelevance and language, which gave 14 studies for full inclusion. These were in majority case reports, but included one literature review and book source as well as two pictorial radiological reviews. These were then further analysed for our outcome measures.

For ease of read, the data for primary outcome measures are portrayed in tables. Out of the 13 patients in this review, only a few were paediatric or elderly patients (Table 1). Young adults were the most common, as were females compared to males. Ethnic origin was not recorded in any of the papers identified.

**Table 1:** Population group breakdown by age group, age range and sex

Population group	Number of patients (n)
Total patients (n)	13
Age group (years)	
Adult	10
Adult (+pregnant)	1
Paediatric	2
Age (years)	
0-20	2
20-65	10
65-90	1
Sex	
Male	2
Female	11

Abdominal pain was observed in every patient and in majority was present in the epigastric and RUQ areas. Most patients presented within 72 hours of the onset of symptoms (Table 2).

**Table 2:** Presenting complaint features divided into symptoms, abdominal pain location and time to hospital presentation

Presenting complaint	Number of patients (n)
Symptoms at presentation	
Abdominal pain	13
Nausea	6
Vomiting	2
Anorexia	1
Abdominal pain location	
Epigastric	10
Umbilicus	1
Right upper quadrant (RUQ)	8
Left upper quadrant (LUQ)	1
Right iliac fossa	1
Time to hospital presentation (hours)	
0-24	2
24-72	6
>72	1
Unrecorded	4

Past Medical and Surgical History

Of all the studies reviewed, only one paper had the patient’s previous medical history included. In addition, the surgical history documented was limited, with two patients having recorded previous surgeries: one had a laparoscopic Roux-en-Y bypass surgery for obesity 10 months prior to their F-FLAT presentation [5], and the other patient had a previous laparoscopic cholecystectomy and vaginal prolapse surgery [6].

Biochemical Markers

Biochemical data was limited: in less than half of the patients (of which we had available data), inflammatory markers were raised, but due to incompletion, valid conclusions were hard to draw (Table 3).

**Table 3:** Patients' biochemical markers WCC, white cell count; CRP, C-reactive protein; UE, urea and electrolytes; LFT, liver function test; NA, not available; n, number of patients Biochemical markers were divided into WCC, CRP, UE, LFTs, and amylase and categorised to see if they were within normal ranges.

	Normal (within normal range), n	Abnormal (outside normal range), n	N/A
WCC	5	5	3
CRP	4	2	7
UE	7	0	6
LFTs	5	1	8
Amylase	5	0	8

Investigations and Diagnostic Procedures

Abdominal US was the first-line investigation for all the studies reviewed except two (Table 4). In these two cases, abdominal CT was done in the first instance. All the cases that had abdominal US done were then followed up with abdominal CT afterwards as the second-line investigation to confirm pathology. In two other cases, abdominal x-ray (to rule out abdominal perforation) and oesophago-gastro-duodenoscopy (to rule out marginal ulcer at a Roux-en-Y anastomosis), were performed additionally.

**Table 4:** Diagnostic investigations CTAP, CT of the abdomen and pelvis; US, ultrasound; N/A, not available; n, number of patients

	Performed, n	Not performed, n	N/A
Abdominal US	9	2	2
CTAP	11	0	2
US + CTAP	9	4	0
Further imaging	2	3	8

Management: Conservative Versus Surgical 

**Figure 5:**
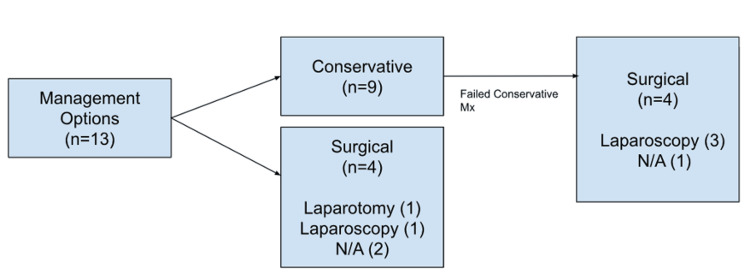
Flowchart depicting the management process and surgical approach for F-FLAT F-FLAT, fatty-falciform ligament appendage torsion; Mx, management; N/A, not available Image credits: Divakar Hamal (first author)

Figure 5 depicts the management course in a flowchart format: out of the 13 patients, nine were managed conservatively with analgesia (including non-steroidal anti-inflammatory drugs, or NSAIDs), and the remaining four were managed with surgery. However, four of the nine patients managed with conservative treatment went on to have surgery due to unresolving pain.

Surgical Follow-Up and Histology Results

All the surgically managed patients had intraoperative samples from the excised ligament sent to pathology. Histology results showed fat tissues, high vascularity and inflammation and necrotic, thrombotic and inflammatory infiltrated cells of the resected tissue. Furthermore, only four studies had a documented follow-up, with two studies reviewing in two weeks post-diagnosis, one study following up eight weeks after and one with no timeframe reported. All four patients in the studies had completely resolved symptoms.

Interpretation of findings

Infarction of the falciform ligament, pathologically defined as F-FLAT, is a rare diagnosis, with only 13 patients having F-FLAT reported in the literature till now. Other types of IFFI such as omental infarction and epiploic appendagitis are more common [7], yet are as important as differential diagnoses and for radiological awareness [4]. As referred to in the Results section, our points of interest were the population group affected, clinical presentation, investigation management and the follow-up.

The falciform ligament is derived embryologically from the ventral mesentery and thus attaches from the ventral abdominal wall and inferior diaphragmatic surface, stretching past the anterior liver margin and across to the posterior visceral surface. This consequently gives a clear demarcation of the liver’s left medial and right lateral lobe. Arterial drainage comes from the middle hepatic arteries and left inferior phrenic artery whereas the left inferior phrenic vein is the sole venous drainage. The ligament’s free edge consists of the ligamentum teres, obliterated umbilical vein (paraumbilical veins) and extraperitoenal fat/appendages, the latter of which can vary in individuals across the whole ligament’s tract, and where F-FLAT is pathologically defined [2]. It occurs when the fat twists and torts, cutting vascular blood supply back to the central vein leading to ischaemia, thrombosis, infarction of the falciform ligament and ultimately necrosis in some cases.

The population group and the clinical presentation were the first area we broke down: we found that over 80% of patients were adult females, but it is difficult to associate the two sexes with the pathology, due to the condition being so rare. The review also illustrated that past medical and surgical history of the patients were scarcely documented, with only three cases giving evidence of them, which makes any specific risk factors almost impossible to correlate and justify. Presentation wise, every case study documented abdominal pain as the primary symptom followed by nausea as the second most common; pain in over half of the cases reviewed was localised to the epigastric and right upper quadrant area. It has been described in the literature that this acute presentation can mimic other surgical pathologies such as appendicitis, cholecystitis and less commonly, hepatitis and bowel perforation [7-9].

Biochemically, there was some evidence of mildly raised inflammatory markers, yet 50% of patients had a normal white cell count and CRP levels. Moreover, both these were documented in only six studies as there were numerous data gaps within the blood results reviewed, further illustrating insufficient data. Imaging is therefore crucial in diagnosing F-FLAT.

Ultrasound typically presents with a hyperechoic mass with adjacent inflammatory changes, heterogeneous in size and noncompressible, deep to the rectus abdominis, has absent colour flow on Doppler and corresponds to the patient's pain on palpation [10,11]. Less than 50% of the cases who underwent ultrasound had one or more of these features, which shows the unreliability of this imaging method as a diagnostic tool, as well as the fact that it is dependent on sonographic interpretation and so can be missed in its entirety [10,12].

Abdominal CT with contrast is much more sensitive with coronal and sagittal views depicting increased density within the fat joining the falciform ligament with associated inflammatory changes as well as sometimes a peripheral rim of hyperattenuation, with the “hyperattenuating rim sign” indicating vascular compromise. Furthermore, one can subsequently see “a central dot sign” that is a hyperdense focus indicating a thrombosed central vein [4]. Out of 11 CT scans, nine reported specific fat stranding of the falciform ligament and three cases describeed the pathognomonic signs mentioned above. The specific pictorial papers were very useful at differentiating the different IFFI and gave us good radiological insights into imaging specifics and diagnostics [7,13,14]. Due to the rarity of this pathology, CT is also very helpful in ruling out other differentials such as omental infarction, as radiologists can reconstruct images in multiple different planes and can therefore distinguish F-FLAT definitively [10]. However, less experienced surgeons or radiologists may miss the diagnosis, especially if only axial views are looked at, so a thorough interpretation of sagittal views is paramount [4].

A conservative management approach to F-FLAT is preferred, yet due to a worsening clinical picture and unresolving abdominal pain, surgical resection might be indicated. Analgesia (NSAIDs/opioids) was the most common conservative method used, and specific antibiotic use was not mentioned in any of the F-FLAT cases we identified. Furthermore, other than the one laparotomy case, surgical techniques and details were not expanded on in the rest of the cases we reviewed, and data were minimal. Due to the condition being so rarely reported in the literature and the limited data available, one cannot compare conservative versus surgical management at first presentation, but can infer a conservative approach is preferable in the initial F-FLAT presentation.

## Conclusions

F-FLAT is a rare and unfamiliar presentation that warrants careful attention to differentiate it from other common acute pathologies that it can imitate. The literature review of this rare condition suggests that F-FLAT occurs more commonly in adult females and always presents with abdominal pain, usually epigastric or in the RUQ region. Biochemical markers are nonspecific, and thus imaging with CTAP with contrast is often required to diagnose this rare condition and also exclude common acute surgical pathologies that F-FLAT tends to mimic. Initial management with analgesics alone is not unreasonable, though surgical resection with a laparoscopic approach where available might need to be done if symptoms do not settle, which could be a marker for necrosis.
